# He Jiankui´s gene‐editing experiment and the non‐identity problem

**DOI:** 10.1111/bioe.12878

**Published:** 2021-05-05

**Authors:** Marcos Alonso, Julian Savulescu

**Affiliations:** ^1^ Department of Philosophy Faculty of Liberal Arts Adolfo Ibáñez University Vina Del Mar Chile; ^2^ Oxford Uehiro Centre for Practical Ethics Oxford University Oxford United Kingdom of Great Britain and Northern Ireland

**Keywords:** CRISPR, future people ethics, gene editing, He Jiankui, non‐identity, procreative ethics

## Abstract

Genetic engineering has been a topic of discussion for over 50 years, but it is only recently that gene editing has become a reality. CRISPR biotechnologies have made gene editing much safer, precise and feasible. We have witnessed the first cases of human germline genetic modification resulting in live births, conducted by He Jiankui. In this paper, we will analyse He Jiankui’s case in relation to one of the most difficult problems in procreative ethics (or the ethics of future generations): the non‐identity problem. We believe that this analysis will help us to understand the ethics involved in gene editing and hopefully allow for a better, more philosophically grounded legislation on CRISPR and other gene‐editing technologies.

## 1 INTRODUCTION

Although genetic engineering cannot be considered a new technology, CRISPR has taken it to a whole new level. The quickness and precision of this new biotechnology, as well as its wide range of applications, make CRISPR nothing less than a revolution.^1^ The history is by now well known: the work of F. Mojica on the immune system of archaea and bacteria in the 1990s served as the basis of the development by J. Doudna and E. Charpentier in 2012, and shortly after by F. Zhang, of a gene‐editing technology that emulated the bacterial immune system’s ability to cut DNA with high precision. Since then, this technology has been successfully applied to all kinds of organisms, including mammals, and has been perfected and amplified not only to cut, but also to add genes. More recently, ‘prime editing’, developed in 2019 by David Liu and his colleagues at the Broad Institute (Cambridge, Massachusetts), seems to have reduced significantly the shortcomings of CRISPR, namely its precision, by making it ‘possible to insert or delete specific sequences at genome targets with less collateral damage’.^2^ Doudna and Charpentier won the Nobel Prize in 2020 for their work on CRISPR.

Gene editing of humans has been controversial since the introduction of this technology. However, many have been surprised by how fast its implementation has been. In 2015, J. Huang and his Chinese team reported that they had edited human zygotes. Two years after, in 2017, K. Niakan’s group in the U.K. was able to genetically modify human blastocysts. But it was in the last months of 2018 that Chinese researcher He Jiankui shocked the world when he claimed to have helped in the birth of the first genetically modified human babies, Lulu and Nana, with a third pregnancy underway.

He Jiankui’s experiment recruited HIV‐serodiscordant couples: couples in which the man is HIV‐infected, while the woman is not. The couples were informed that this procedure might make their children immune to HIV, and had their fertility treatment and pregnancy medical care paid for by the researchers. During IVF, the sperm was ‘washed’ from the semen.^3^ It is estimated that sperm washing reduces the risk of HIV transmission from the father to almost 0%.^4^

Embryos were created by placing one sperm of the male participants into one egg of their female partners. Some of these embryos were edited using CRISPR, and couples were offered to choose from edited or unedited embryos.^5^ Dr. He Jiankui’s intervention aimed to change both copies of a gene named CCR5, with the alleged intention of making the white blood cells of the future babies unable to get infected by HIV. There is also some suspicion that CCR5 modification is related to brain development and therefore to cognitive enhancement.^6^

The experimental outcome was far from clean. CRISPR modified each of the twins differently: in one twin, CRISPR modified only one of the two chromosomes, whereas in the other, it altered both chromosomes. Also, in both twins not all the cells had modified CCR5 genes, making them ‘mosaics’.^7^ As germline edits, these gene modifications might get passed to Lulu’s and Nana’s descendants. It is also worth noting that He Jiankui’s experiments resulted in the birth of a third gene‐edited baby during 2019. We do not have details about this third baby.

The pace of gene‐editing trials on humans has been widely criticized. But the repudiation of Jiankui’s experiment was on another level, generating an almost unanimous backlash and receiving criticism for its insufficient safety measures, its scientifically unsound methods, its lack of ethical supervision, and its suspected hidden motivations.^8^ Legal actions have been taken against He Jiankui. The Chinese doctor was found guilty in December 2019 of ‘illegal medical practices’ and sentenced to three years in prison.

Many of the criticisms directed at his experiment seem justified, although we still lack precise information about it. However, there has not been a sustained philosophical analysis of the philosophical issues involved in such gene editing. In this paper, we will analyse He Jiankui’s case in relation to one of the most difficult problems in procreative ethics (or the ethics of future generations): the non‐identity problem. We believe that this analysis will help us to understand the ethics involved in gene editing, and hopefully allow for a better, more philosophically grounded legislation on CRISPR and other gene‐editing technologies.

## 2 HE JIANKUI’S CASE AND THE NON‐IDENTITY PROBLEM

Prior to He Jiankui’s experiment, the world saw a similar case that did not gain nearly as much attention. In September 2016, a baby was born in Mexico after Dr. John Zhang, a New York City‐based fertility doctor, performed a mitochondrial replacement procedure. This procedure may arguably be considered itself a form of genetic modification. However, setting this discussion aside, what is interesting for present purposes is the justification Dr. Zhang gave for carrying out this intervention. When asked why he conducted the mitochondrial replacement procedure, he simply responded: ‘To save lives is the ethical thing to do’.^9^ Similarly, in the video He Jiankui posted on YouTube, he emphasized the importance of Lulu’s and Nana’s health and how decisive the gene ‘surgery’—as he described it—was to protect Lulu’s and Nana’s lives.

As we noted, these interventions have faced criticism for their lack of safety, supervision, and appropriateness.^10^ But here we want to ask a more fundamental question. Did Dr. Zhang and Dr. Jiankui really save, cure, or harm anyone, even if their procedures were successful? Both procedures involved IVF and the creation of new life. It seems that the children that were born after the interventions would not have lived otherwise, if the procedure had not been performed, which makes it difficult to identify whose life was saved, harmed or cured in these cases. It seems that we are facing what has been called a non‐identity problem.

The non‐identity problem, developed in D. Parfit’s *Reasons and Persons* (1984), is one of the central problems of the ethics of future generations. While most ethical problems concern actions that violate the rights or decrease the well‐being of existing people, some actions also determine the existence, number or identity of future people. The peculiarity of these actions is that, strictly speaking, they cannot benefit or harm any individual, as they are the cause of their very existence.^11^ To better understand this point, we will use a paradigmatic example: Parfit’s pregnancy case. This case is reformulated by Boonin as follows:

Wilma has decided to have a baby. She goes to her doctor for a check‐up and the doctor tells her that there is some good news and some bad news. The bad news is that as things now stand, if Wilma conceives, her child will have a disability [the child will be born incurably blind]. The good news is that Wilma can prevent this from happening. If she takes a tiny pill once a day for two months before conceiving, her child will be perfectly healthy. The pill is easy to take, has no side effects, and will be paid for by her health insurance. Fully understanding all of the facts about the situation, Wilma decides that having to take a pill once a day for two months before conceiving is a bit too inconvenient and so chooses to throw the pills away and conceive at once. As a result of this choice, her child is born with a significant and irreversible disability.^12^

When presented with this case, most people believe that Wilma has done something morally wrong. Most people think that, even though her child will presumably have a life completely worth living, Wilma harmed the child with her decision to not wait and take the pill. But the problem is that, had Wilma waited, the child who would have been born would be a different one, as the sperm and egg that would form the embryo would be different (to reinforce this idea, Boonin names the blind child Pebbles, and the sighted child Rocks, also giving them different sexes). However, if this is the case, we face the paradox that Pebbles has not been harmed in any way and, for this reason, Wilma has not done anything morally wrong, even if we intuitively feel she has.

The non‐identity problem is central to many bioethical problems. As Roberts and Wasserman point out, ‘the nonidentity problem represents a challenge not just for moral philosophy but also for law and public policy’.^13^ Boonin thinks likewise, declaring that the non‐identity problem, ‘despite its esoteric appearance, has a direct bearing on a number of important moral dilemmas that confront us in the real world’.^14^ The non‐identity problem has implications for cloning, IVF and other reproductive technologies and germline genetic modification, as we will now explore.

## 3 THE NON‐IDENTITY PROBLEM IN GENE EDITING?

The non‐identity problem typically arises when we are considering selecting between two possible persons, such as selecting which embryo to implant. A well‐rehearsed case is one where we are selecting between a hearing and a deaf embryo. If one selects the deaf embryo, one has not harmed the future person, because that individual would not have existed without being deaf.^15^ If this is wrong (which we believe it is), it is wrong in an impersonal rather than a person‐affecting sense.^16^

However, when one changes the genes for better or worse of an existing embryo, this typically causes a person‐affecting harm. For example, if one were to have a deaf baby, Jane, by taking a normal embryo and deleting the genes for the development of normal hearing, one would have harmed Jane, just as if we had cut Jane’s auditory nerves soon after birth.

He Jiankui has been criticized for exposing Lulu and Nana to unreasonable risks of harm.^17^ For example, Raposo explains that because ‘gene editing was incomplete in at least one of the babies (…) some issues for the children's future health may [have] resulted from the outcomes of this procedure’.^18^ Studies have shown that CCR5 genes deficiency can lead to a higher risk of infection from the West Nile virus and severe flu.^19^ Chinese bioethicists have also emphasized the uncertainty on ‘whether the genetic intervention made them [the girls] more susceptible to other infections or affected their health in other ways or what effect it will have on their children and their children’s children’.^20^ These are person‐affecting harms. If Lulu or Nana get cancer in the future, they could rightly complain that they were harmed by the gene editing, which caused the cancer. They could have existed without the CCR5 deletion (introduced by Jiankui to confer resistance to HIV) and the associated off‐target mutation. So it does not appear that gene editing raises issues of non‐identity. However, closer scrutiny shows that the situation is more complex in two ways.

First, if gene editing alters brain development in a way that causes a significantly different psychological trajectory, it would be identity‐altering. It would be identity‐altering if it caused a different chain of psychological connectedness and continuity on a psychological or narrative account of identity.^21^ Persson rejects this interpretation,^22^ but other authors, such as McMahan, think that interventions of this kind can be identity‐determining.^23^ Thus, if the deletion of CCR5 or off‐target effects caused significant intellectual disability, they would be identity‐altering.^24^ As we have mentioned, CCR5 has been implicated in brain development.

Secondly, the creation of Lulu and Nana could be identity‐altering in another way. Although once created the embryos that gave rise to Lulu and Nana could have existed without the gene editing, and so any harm could have been avoided, the creation of those embryos was dependent on He Jiankui’s actions. The parents of Lulu and Nana could not access IVF because they had insufficient funds to pay for the procedure themselves. The father had HIV. If they had conceived naturally, a different child would have been born with a substantial risk of contracting HIV from the father.^25^

He Jiankui employed antiretrovirals to lower the viral load of the father. He also organized and paid for the sperm to be washed clean of HIV. So a different child was born, who was free of the risk of HIV. Therefore, in one sense, He Jiankui’s funding of an experiment to create embryos by IVF (and protect them from HIV by sperm washing) was identity‐altering. We can call this Policy‐Related Identity Alteration (PRIA).

One might be suspicious that PRIA is identity‐altering in the same way as Parfit’s original examples. For example, a deaf couple might have a 50% chance of having a deaf child by natural conception. They wish to have a deaf child. So they engage in IVF but only produce hearing embryos. They seek to gene edit a hearing embryo to make it deaf. ‘If it were not for the IVF, this embryo would not exist, and without the gene editing we would not have implanted it. So it has not been harmed because the IVF and gene editing is PRIA.’

This may be a case of PRIA, but it is an unethical case—there is no good reason to create an embryo to deafen it. Doctors could legitimately say, ‘We are not going to provide IVF and gene editing so you can deafen a child’.

We believe that the case of He Jiankui is different. There was a good reason for Jiankui to use IVF and sperm washing: to protect the embryos from contracting HIV from the father. And given that Jiankui was using private funds and was under no obligation to use those funds to provide Good Samaritan IVF and sperm washing, it may have been reasonable to tie this to additional gene editing as a part of scientific research. There was a reason in favour of his research, and a reason against. Those reasons must be weighed. It depends on whether the risks and expected harm of contracting HIV from the father were greater than the risks and expected harm of the off‐target effects. If they were greater, it may have been an example of permissible PRIA. We will proceed on the assumption that the gene editing of Lulu and Nana was an example of PRIA. (Whether it is justifiable is a related but further issue.)

## 4 DIFFERENT APPROACHES TO THE NON‐IDENTITY PROBLEM AND THEIR ASSESSMENT OF HE JIANKUI’S CASE

To understand the complex and widely discussed non‐identity problem, we will consider some of the most important proposals that have been elaborated to solve it. Then we will show how each of these solutions applies to He Jiankui´s case.

### 4.1 Impersonal solutions

Many authors have sought to solve the non‐identity problem by denying the idea that harm and moral wrongness must necessarily be person‐affecting.^26^ They accept that the baby or babies created—Pebbles in Wilma’s case, Lulu and Nana in Jiankui’s case—were not harmed; but nonetheless they defend that some form of impersonal harm and wrong has been done. The bottom‐line idea is that a better world could have been generated, and failing to do so was wrong. On this view, ‘a wrong act is, in at least some cases, wrong not in virtue of whether it makes things better or worse for particular persons but rather in virtue of its failure to promote or secure certain “impersonal” values, ideals or effects’.^27^

In the case we are trying to assess, the impersonal solution of the non‐identity problem indeed matches the general consensus that He Jiankui did something wrong with his experiment. From an impersonal point of view, the Chinese researcher did not harm anyone, but it could still be argued that he did something wrong by not selecting an embryo that would have developed into a child who would have benefited more from the intervention (for example, selecting an embryo with the genetic disorder Tay–Sachs disease).^28^ He Jiankui failed to create the best possible world he could have created.

This line of reasoning is characteristic of consequentialism, a philosophical theory that focuses on maximizing happiness, well‐being, or other form of good. However, this way of thinking, when it is not qualified in some degree, results in much more counterintuitive conclusions than the ones derived from the non‐identity argument. A fully coherent consequentialist position would force us to defend that the decision to not reproduce, at least when we know the new lives created would be full of well‐being, would be morally wrong. This way, we would face an ‘unconstrained obligation to procreate’.^29^ We would also be forced to agree with the idea that having blind children is, in some respect, immoral. And we would also be committed to the claim that a large enough number of miserable lives would be better than a low number of great lives.

In He Jiankui’s case, we can see how the impersonal solution to the non‐identity problem, even if it matches well our intuitive assessment of the wrongness of this experiment, carries many unpalatable conclusions. For example, the impersonal solution could entail that He Jiankui ought to have developed as many embryos as he could. Instead of choosing between embryos, Jiankui should have implanted as many embryos as possible. If the human lives emerging from these embryos were expected to have a life worth living, this proliferation of lives would mean bringing more well‐being to the world and thus create a better world. This seems a counterintuitive imperative. The impersonal position might be also forced to accept that if He Jiankui contributed to the birth of a sufficient number of healthy, non‐edited babies, the possible diseases and problems of Lulu and Nana would be outweighed by the net sum of happiness or well‐being created.

Some philosophers have tried a moderate, narrower version of this proposal. For example, Parfit’s ‘Principle Q’ states that if ‘the same number of people would ever live, it would be worse if those who live are worse off . . . than those who would have lived’.^30^ Parfit does not want to completely avoid the reference to persons, as he believes—rightfully so, in our opinion—that common sense morality is so rooted in person‐affecting views that postulating a completely impersonal morality would be way too incomprehensible. By adding the ‘same number’ clause, he seems to avoid the most abhorrent conclusions of extreme consequentialism. However, Parfit himself was never fully convinced about this solution, and indeed it seems to face some important problems of its own.

According to Q, He Jiankui should not have gene edited Lulu and Nana, because then they would have existed with worthwhile lives, without the risk of off‐target mutations. However, as we have argued, He Jiankui was not under an obligation to provide IVF and sperm washing to this couple. If he had decided not to conduct his experiment, Lulu and Nana would not have existed. It is, in one sense, not a same‐number situation.

The alternative would have been for the parents to conceive naturally. This would have exposed their children to a high chance of contracting HIV from the father. So if it were a same‐number situation, He Jiankui may well have brought about the best outcome, even with gene editing.

Even if we restrict to same‐number cases, the objection to He Jiankui’s case posed by the impersonal solution is substantially weaker than the general condemnation his experiment received. The impersonal solution to the non‐identity problem cannot establish that He Jiankui’s experiment was *fundamentally* wrong, as far as it involved PRIA. It did not create a life absolutely not worth living. In fact, from its perspective, carrying out the experiment was a *fundamentally good* thing to do, as it helped to create life that will expectedly be worth living. The criticism from the impersonal solution is only that the benefit could have been greater. Also, assuming this position might force us to criticize the birth of normal individuals, when an enhanced individual could have instead been created. Even if a positive prescription to have the best possible children seems defensible,^31^ it seems excessive to extend this kind of prescription and criticize the creation of worthwhile lives, as Lulu’s and Nana’s expectedly will be.

### 4.2 Rights‐based solutions

One of the other main ways in which authors have tried to solve the non‐identity problem is by accepting the person‐affecting clause, but rejecting the premise of the non‐identity problem that affirms that no one was wronged. The point of these proposals is that, even though we cannot say that the person created was harmed, that person was not treated up to the appropriate standard. These types of arguments affirm that in non‐identity cases we fail to treat a person as an end‐in‐itself, we do not make sure our procreative acts are virtuous, or we do not fully respect a person’s rights. We will focus on the last strategy, the so‐called rights‐based solution to the non‐identity problem, which seems the most promising within this area.

Magnusson states that the key of this rights‐based proposal is understanding that ‘rights do not attach to people’s numerical identities, but rather to the kinds of creatures they are, and the positions they occupy in relation to others’.^32^ Thanks to this specification, the fact that the affected individual did not exist when the action took place becomes irrelevant for its wrongfulness. Independently of the subject’s existence, a right was violated, and that is what deems the action wrong.

Looking at He Jiankui’s case, embracing the rights‐based solution would imply criticizing He Jiankui’s experiment for ignoring or even undermining Lulu’s and Nana’s rights. From a rights‐based perspective, it could be said that He Jiankui harmed Lulu and Nana by not respecting their genomic integrity. It could also be said that He Jiankui’s intervention constituted an attack on Lulu’s and Nana’s future autonomy, severely damaging their right to have a free and unfettered life.

Again, this line of reasoning seems promising, but it is not immune to objections. The main problem is that the rights‐based solution seems so rigid that it results in undesirable authoritarian conclusions. Rights‐based solutions would seem to imply that the rights violation is so strong that it is not outweighed by the fact that the individual created would otherwise not enjoy any of the goods in that person’s life:^33^ a conclusion that, again, seems quite implausible and counterintuitive. A case that further exemplifies this is the following:

Suppose, for example, that you will soon die if I don’t donate a kidney to you, and that I can either promise that I will give you a kidney and promise that I will paint your house, in which case I will keep the first promise but break the second promise, or that I can promise to do neither, in which case I will do neither. In this case, it seems clear that it would not be wrong for me to make both promises and then keep the first one, even though doing so would again generate in you a right that would later be violated.^34^

In this scenario, it seems clear that anyone would happily ignore their right to have their house painted in favour of the great benefit of staying alive. A closely related line of argumentation has been the exploitation solution to the non‐identity problem. This type of solution responds to the waiving of rights objection by exposing that this kind of reasoning would resemble exploitation cases in which, for example, a mean factory owner offers an extremely badly paid job. A worker otherwise unemployed is better off with that miserable job, but that does not mean that something wrong is not happening. While this reasoning also holds a good amount of intuitive truth, it is also subject to criticism. Boonin comes up with an illustrative counterexample:

Suppose, for example, that Ann will soon die if she does not drink some water, that the only person who can provide her with the needed water in time is Keith, and that Keith knows that Ann’s net worth is a little over two million dollars. And suppose that Keith offers to give Ann the water only if she gives him two million dollars and that Ann accepts.^35^

In this case, we are inclined to say that Keith exploits Ann because he takes advantage of her vulnerability, extracting from her a price that is unfairly high. However, the difference between this case (as well as the case of the mean employer and other exploitation cases) and procreative cases is that the unfair offer could easily be changed into a fair one: Keith could charge Ann a fair price, and the employer could pay a fair salary. In the case of Lulu and Nana, they would not have existed were it not for the experiment—another child at risk of contracting HIV from the father would have existed.

Again, similarly to what happened with impersonal solutions, the consequences of a rights‐based solution prove to be at least equally counterintuitive as accepting the non‐identity argument’s conclusion. A coherent defence of rights could force us to defend extremely restrictive procreative policies. Any less than perfect child, or any less than perfect conditions of birth, would amount to a rights violation, limiting reproduction to the wealthiest and healthiest couples.^36^ This, again, seems highly counterintuitive and problematic.

#### 4.2.1 Non‐comparative harm solutions

Somewhat related to rights‐based solutions, at least in their common acceptance of the ‘person‐affecting’ clause and their denial of the ‘no‐harm’ clause, are non‐comparative harm solutions. Advocates of these proposals^37^ argue that harm should not be understood comparatively, but substantively. From this point of view, harm is a discrete reality that comes into existence when someone is subject to non‐comparatively harmful states, such as ‘pain, early death, bodily damage, or deformity’.^38^ This line of reasoning does not deny that, in some cases, benefits could outweigh the harms; but even in those cases, we must admit that harm was done. In Lulu and Nana’s case, this could mean that bringing them into existence harmed them, even if bringing them into existence also benefited them.

If we assume all of the above, we could say that He Jiankui’s actions were wrong from a non‐comparative harm solution view in two ways. The first way, which we could call the overall non‐comparative harm version, would say that harm is done if the overall benefit–harm ratio was in favour of harm. Even if we still do not have complete information about the case, it does not seem that harm done to Lulu and Nana outweighs the benefits of ever existing. From what we know, and even if it is very difficult to measure, the risk of early and severe harm in the form of disease or deformity does not appear to be too high, at least not in comparison to other cases that medical practice generally allows. But even if the worst‐case scenario happened and some early and severe genetic disease manifested, Lulu and Nana would still have experienced some of the goods of life, what it is to play and have fun, to have a good meal and to feel the love from their family. This seems like an overall acceptable trade‐off, particularly when we understand that the alternative is that they do not exist at all, on PRIA. Only an extremely short and painful life, like the one involved in Tay–Sachs disease, could plausibly outweigh the benefits. That does not seem to be Lulu and Nana’s case. A finer comparison and discussion might be in order here. But we would say that, in a case of low probability of harm and high probability of benefits, only some kind of extreme precautionary principle would deem such a case wrong. It is likely that overall the expected benefits of such a life would outweigh the expected harms, and so their lives would be overall worth living.

There is a second way in which we could consider the non‐comparative harm done to Lulu and Nana. This second way, which we could call the discrete non‐comparative harm version, would not compare benefit and harm in equal terms, but would take into account the idea that not harming is more important than benefiting. This seems to be a plausible intuition, and, as Harman argues, ‘reasons against harming have stronger force than reasons to benefit’.^39^ This is the idea behind the well‐known medical principle of ‘non‐maleficence’, and it is based on a generally widespread preference of avoiding harm over obtaining positive pleasure.

One example might add force to this reasoning. Arguably, most people would allow their little finger to be cut off in exchange for 10 million dollars. But that does not make it permissible for Jeff Bezos to be cutting people’s little fingers off indiscriminately, even if he would pay 10 million dollars afterwards. Cutting fingers without permission is harmful, and therefore wrong, despite the benefits. However, this can be disputed. What if cutting the fingers was not an indiscriminate action, but its intended purpose was benefit? It is true that this example would need a very farfetched explanation, saying, for instance, that Bezos had gone crazy and believed that little fingers were the origin of many important diseases. But if we could believe that this person is truthfully convinced, and that his intention when cutting fingers was to benefit, would we still think his actions were wrong? He produced harm, but the intended goal was benefit, and the 10 million dollars he gave to each injured person seems like a very significant benefit.^40^

If we turn again to He Jiankui’s case, the comparison is even more favourable, as the couples entered the experiment with information about it disclosed beforehand. If this is so, we must ask ourselves: how much of our rejection of his actions comes from the difficulty in believing that he was intending to benefit rather than, say, to gain fame and fortune? If we accept, just for the sake of argument, that in fact He Jiankui wanted to benefit Lulu and Nana, can we still say he did something wrong? From the discrete non‐comparative point of view, it is clear that he harmed them. But is that enough to deem his acts to be wrong?

Harm can be outweighed by benefit. Saying otherwise would bring many unacceptable implications. Harman herself admits this point, and simply states that harm is harder to outweigh than benefit. However, we might ask, how much harder? If it is a matter of degree, then the non‐comparative harm solution does not, properly speaking, solve the non‐identity problem, it just gives us a finer‐grained comprehension of an aspect of it. If it is not a matter of degree, we might just be staring at a standard rights‐based solution, with the problems mentioned above; particularly, that any less than perfect child, or any less than perfect conditions of birth, would amount to a rights violation.

There is another important issue. Even if the preceding argument supporting the non‐comparative harm interpretation seems plausible, it is not clear that non‐identity cases can be perfectly fitted into its scheme. The overall non‐comparative version of substantive harm seems to put a high enough threshold such that many of the non‐identity cases would not be affected. Is blindness, for example, a form of substantial harm? If it is, it could imply, at least in the overall non‐comparative version of substantive harm, ‘that there is something intrinsically bad about the world containing blind people’, implying ‘that every time a blind person is born, the world becomes a worse place, that it would have been better, from the moral point of view, if such people had never existed’.^41^ This is a conclusion extremely hard to believe. Within the discrete non‐comparative harm version, it could be argued that parents who have blind children harm them; however, it seems very difficult to defend that this harm makes the action of having a blind child wrong. Only a position that privileged not harming absolutely above benefiting could propose so. But such a position would have even more problematic implications.

### 4.3 Acceptance solutions

Other solutions to the non‐identity problem have been proposed, although the main ones have already been covered. The only one remaining is the simplest one: disregarding the non‐identity problem as a problem and accepting its argument, including the counterintuitive and discomforting conclusion that no harm was done in bringing a new child to life. As Narveson famously said, ‘we are in favour of making people happy, but neutral about making happy people’.^42^ This acceptance solution has been defended by some authors.^43^ The acceptance solution seems, on first instance, a cheap way to bypass the problem itself. Its point, however, is that it may very well be that our intuitions regarding this problem are flawed and the non‐identity’s conclusion is not so implausible as we tend to think when we first encounter it. As Boonin states, if after careful consideration you cannot find a convincing flaw to an argument, it is ‘more reasonable for you to simply accept the argument’s conclusion than to continue to reject the argument simply because you initially assumed that the argument’s conclusion was mistaken’.^44^ He gives the following analogy:

Suppose that Peter comes home from work and finds two envelopes in the mail. The first contains an unexpected windfall: a check for fifty dollars. The second contains a letter from Oxfam correctly and convincingly informing him that if he contributes fifty dollars to their organization, one less child will die prematurely from hunger or disease. After giving it some thought, Peter throws the letter from Oxfam away and instead spends the fifty dollars on dinner and a movie.^45^

The reason to bring up this example is not to show that Peter’s act was morally impeccable, but to show that most people find it plausible that Peter’s act was not morally wrong. After all, many of us find ourselves in situations of this kind, in which we could use our resources to greatly benefit others, and we do not. And the point is that, as Boonin argues, ‘even those who argue that it is positively immoral for Peter not to contribute the money to Oxfam under such circumstances clearly and explicitly recognize that they are arguing against the commonsense view of the matter’.^46^ Which, again, is enough to show that it is plausible to think that Peter’s act was not morally wrong.

Or consider another analogy. A man is in a raft down a rapid river with his wife and two children. The raft overturns. His wife is swept down one fork, his children down the other fork. There may be no singular right answer regarding whether he should rescue his wife or his two children.^47^

Returning to He Jiankui’s experiment, we must now ask ourselves: how would the acceptance position assess this case? If we understand Lulu and Nana’s case as a non‐identity case (at least as PRIA), we must conclude that Jiankui did not harm them in any way.^48^ Given that their lives are worth living, bringing them to existence did not harm them. Properly speaking, from this perspective, bringing them to existence *could not* have harmed them if their lives are worth living, as the only alternative would be to have never existed.

### 4.4 Another solution: Indirect forms of person‐affecting harm

However, can we? Can we really say that *nobody* was harmed? The impersonal and rights‐based views already seemed to diminish the perceived wrongness of He Jiankui’s experiment. However, the acceptance solution goes several steps further and simply states that no one was harmed, and that, arguably, nothing wrong happened in regard to this experiment. This conclusion goes against most people’s intuitive response to this case, and even if this intuition stands firm, we feel that this is far from being a satisfactory solution.

Furthermore, accepting the non‐identity argument would seem to set the bar way too low and to possibly open the door to a virtually endless array of gene modifications—modifications that would only need to meet the criteria of creating a life worth living to be acceptable. From this perspective, He Jiankui’s experiment would not only have been permissible, but could even be considered a rather prudent and conservative one. Any researcher would, in principle, be allowed to edit embryos in even more extensive and profound ways, provided those embryos would not otherwise exist and have lives which are worth living. For example, under this account, it probably would be permissible to create an embryo deliberately modified to develop blindness, have a significant mental impairment and be deaf, if the embryo would not otherwise be created. Even though it could, again, be argued that nobody is being harmed, just as nobody is harmed in genetic selection, it seems too much to swallow.

We cannot delve into this problem now, as it would require a different article on the subject. However, we can mention that, in fact, the correct framing of this problem probably requires us to broaden the scope and not focus on direct person‐affecting harm. When we say that, if Lulu and Nana’s case is a non‐identity problem, then He Jiankui’s experiment did not harm anyone, we are simplifying the matter. The experiment could still harm many persons in various ways, as we think is the case. First, it could hurt the parents. Having their children with less than optimal health conditions could severely diminish the parents’ well‐being. The experiment could also greatly harm other researchers, who could see their ability to pursue their work hampered owing to the backlash against gene editing occasioned by the actions of Dr. Jiankui. Or He Jiankui’s example may open the floodgates for other reckless experiments, in which identity‐affecting harm could take place.^49^ Other possible impacts could be mentioned. However, the point is that all our previous analysis seems to indicate that the direct person‐affecting harm implicated in the non‐identity problem might not be the main problem of He Jiankui’s case and similar cases. The main claim in this regard has been that He Jiankui’s intervention very possibly ‘made “off‐target” changes elsewhere in the girls’ genomes. Those changes could cause cancer or other problems”.^50^ George Church, one of the very few advocates of He Jiankui, also pointed to harm as the main problem of the experiment, saying that ‘if it had gone south and someone had been damaged, maybe there would be some point [to all the criticism He Jiankui received] (…) That’s probably what it [the experiment’s wrongness] boils down to’.^51^ If direct person‐affecting harm is not the main issue of He Jiankui’s gene‐edit experiment, then our paper importantly shows that the ethical assessment of this case has been generally misguided by focusing on the direct harm done to Lulu and Nana.

## 5 IS HE JIANKUI´S CASE REALLY A NON‐IDENTITY CASE?

To conclude, we must return to the very beginning and examine our first assumption: that He Jiankui’s case is a non‐identity case. The point is that, in Lulu and Nana’s case, Jiankui did introduce the gene edit, and it was in theory possible to have had a non‐edited Lulu and a non‐edited Nana.

To understand this point, it can be useful to briefly go through the discussion of two mitochondrial replacement techniques: maternal spindle transfer (MST) and pronuclear transfer (PNT).^52^ Wrigley et al. argue that MST would, in most cases, be identity‐affecting, because the procedure carried out on the egg would take place before fertilization, causing a different sperm to fertilize the egg than the one that would have fertilized it if there was no procedure. In contrast, with PNT, ‘intervention happens after fertilization, and the gametes used are unaffected, and so the Non‐Identity Problem does not arise’.^53^ But as Rulli has pointed out, it is a somewhat specious portrayal of the real situation. As this author argues, ‘PNT does not treat an existing person or a person who is on its way into existence. Rather the child will exist just because PNT is selected to be used in her creation’.^54^ The point is that, ‘if PNT is not used, there is no child on its way to existence who will be harmed’.^55^ Outlining it as a comparison between PNT and normal conception is an artificial scenario, because ‘real life is nothing like this’.^56^ Thus PNT is identity‐determining in the relevant sense.

Following Rulli’s reasoning, it could be argued that the gene editing of Lulu and Nana was not contingent, but necessary for their existence. This assessment depends on how we view the case. If we hypothetically put ourselves one second before the gene edit took place, we can imagine that He Jiankui could have refrained from editing the embryo and simply introduced the embryo into the mother’s womb, after which non‐edited Lulu and non‐edited Nana would have been born. Even if this does not seem very likely, is not unconceivable. One could imagine Lulu and Nana’s parents having a change of heart and asking the researcher not to edit the embryos, going on to select Lulu and Nana’s non‐edited embryos. We could also imagine He Jiankui realizing the experiment’s ethical problems midway (he has an ethical ‘Damascus Road experience’), leaving the couples free to have their unedited embryos.^57^ Or imagine the Chinese authorities discovering what was going on just before the gene edit, and putting an end to the experiment but allowing the couples to have the embryo they wanted implanted.

These possible scenarios are, however, of dubious moral significance. This counterargument would state that situating ourselves in the second before the gene edit took place is a specious move and brings an ‘artificially contrived scenario’.^58^ The IVF that allowed Lulu and Nana to exist and the gene edit were tied one to another. As Rulli argued regarding mitochondrial replacement techniques, the non‐identity problem does not disappear because there is an imaginable counter‐case. We must argue taking into account reality and likely practice. IVF, and generally egg and sperm selection, is only carried out for special reasons. It is not valid to imagine a case where a couple would undergo IVF when they could conceive the old‐fashioned way without problems. Thus it could be said that the gene editing was a necessary condition of Lulu and Nana’s existence, because if Jiankui was not going to perform the gene edit, he would not have carried out the experiment: he would not have carried out the IVF, and the specific embryos from which Lulu and Nana were created would not have existed (or the chances of them ever existing would have been vanishingly small).

## 6 CONCLUSIONS

If we return to our main question, can we say that He Jiankui harmed anyone? As we saw, this depends on the philosophical problem known as the non‐identity problem. If we understand Lulu and Nana’s case as a non‐identity case (PRIA), we must conclude that Jiankui did not harm them in any way. To deny this conclusion, we may be forced to affirm that the experiment violated a right, or caused a discrete harm, so grave that it would be better if Lulu and Nana had never existed (rights‐based, non‐comparative harm solution). Or, alternatively, we may be forced to defend that Lulu and Nana’s birth was wrong because He Jiankui should have tried to create a quintuplet pregnancy, instead of a twin pregnancy (impersonal solution). However, if we accept the non‐identity argument, and simply declare that no one is directly harmed in He Jiankui’s experiment, this could become a slippery slope that might make permissible an extremely wide range of gene modifications.

As Woollard concludes, ‘perhaps any proposed solution to the Non‐Identity Problem will have to deal with an inevitable lingering dissatisfaction’.^59^ As McMahan admits, ‘Problems in the morality of causing people to exist seem to me the most difficult and intractable of all the problems of which I am aware in normative and practical ethics’.^60^ As Rulli also ends up pointing out, we must be cautious when trying to draw firm and indisputable conclusions from the non‐identity problem.^61^ This does not mean, however, that our previous analysis is pointless. It is useful in pointing out the difficulty inherent in these problems, allowing us to differentiate the strong, unequivocal ethical pitfalls from the problematic issues that are much less clear‐cut and demand a finer‐grained assessment. However, we believe that our paper does contribute in several concrete and important ways to the discussion.

First of all, we have provided insight into a very important philosophical issue that directly affects the ethical assessment of reproductive technologies and gene editing. Analysing He Jiankui’s controversial case has shown that some of the criticism might be considered too harsh or unwarranted. We have many doubts about He Jiankui’s methods and motivations, which, as everything seems to indicate, were wrong and reprehensible. But it is paramount to understand why exactly something is wrong, even if we intuitively and firmly believe so. In this regard, it is crucial to distinguish between the aspects of his experiment that can be deemed as indisputably wrong, and the aspects that cannot be condemned—or less clearly so. This distinction is crucially important to prevent a general negative backlash towards gene editing, a backlash that could paralyse or even set back gene‐editing research and its implementation.

Second, even though the non‐identity problem is a well‐known problem among ethicists, it remains a very complicated problem, which is far from solved. It is also an ethical problem with applications that will increase in the coming years. By analysing this prominent case we have brought to attention a somewhat disregarded problem of the non‐identity literature: the difficulty in defining what constitutes a non‐identity problem in practice. While He Jiankui’s actions may obviously seem to harm Lulu and Nana, more detailed examination makes this much less clear. Sometimes policies or whole chains of actions can be identity‐altering.

Third, with our analysis it became clear that perhaps the most reprehensible aspect of He Jiankui’s experiment is not the harm done to the children (which, as we showed, is either non‐existent or much less grave than is commonly thought), but the harm done to the parents, harm done to other researchers, or harm done to future subjects of experimentation. We believe our analysis significantly enriches the debate by showing that other direct, person‐affecting, harm is at stake.

All in all, we think we should view He Jiankui’s case in a wider perspective. In many parts of the world, including the Western world, couples are free to knowingly conceive a child with a severe intellectual disability or who has a 50% chance of developing cancer. Reproductive freedom is such that it is permissible to bring into existence a child who would not otherwise exist with a certain, or highly likely, chance of having a major illness. Lulu and Nana’s parents chose to bring into existence two children who would be protected from contracting HIV from their father, who also might have lifelong immunity to HIV and some disposition to genetic mutations that might cause serious disease (off‐target effects).

It does seem inconsistent to so harshly condemn one and not at all condemn the other. At the time of the announcement of the news of the birth of Lulu and Nana, it was also announced that there was a third pregnancy under way. No one suggested that the harm was so significant that this pregnancy should be terminated. Indeed, that baby is apparently now born. Reproduction inherently involves risks. If the non‐identity problem offers any justification for not lowering these risks when they can be, it might justify some kinds of gene‐editing experiments.

As one of us has argued, if one were to select an embryo with a catastrophic genetic condition, such as Tay–Sachs disease, and conduct a gene edit on it, this might be an acceptable risk. The fact that this embryo could not be harmed on many solutions to the non‐identity problem adds further support to such experimentation.

## Footnotes

^1^

Carey, N. (2019). *Hacking the code of life: How gene editing will rewrite our futures*. London, U.K.: Icon Books, p. 5.

^2^

Cohen, J. (2019). Prime editing promises to be a cut above CRISPR. *Science (N.Y.), 366* (6464), 406.10.1126/science.366.6464.40631649173

^3^

Li, J., Walker, S., Nie, J., & Zhang, X. (2019). Experiments that led to the first gene‐edited babies: The ethical failings and the urgent need for better governance. *Journal of Zhejiang University‐SCIENCE B, 20*(1), 32–38.10.1631/jzus.B1800624PMC633133030614228

^4^

Zafer, M., Horvath, H., Mmeje, O., Van Der Poel, S., Semprini, A., Rutherford, G., & Brown, J. (2016). Effectiveness of semen washing to prevent human immunodeficiency virus (HIV) transmission and assist pregnancy in HIV‐discordant couples: A systematic review and meta‐analysis. *Fertility and Sterility, 105*(3), 645–655.e2.10.1016/j.fertnstert.2015.11.028PMC477971026688556

^5^

Greely, H. T. (2019). CRISPR’d babies: Human germline genome editing in the ‘He Jiankui affair’, *Journal of Law and the Biosciences, 6*(1), 111–18310.1093/jlb/lsz010PMC681394231666967

^6^

Regalado, A. (2019). China's CRISPR twins might have had their brains inadvertently enhanced, *MIT Technology Review* (Feb. 21, 2019). Retrieved from https://www.technologyreview.com/s/612997/the‐crispr‐twins‐had‐their‐brains‐altered/ [accessed Jan. 29, 2020].

^7^

Greely, op. cit. note 5.

^8^

Carey, op. cit. note 2; Greely, op. cit. note 5; Brave new dialogue. (2019). *Nature Genetics, 51*, 365. Published online: 28 February 2019. 10.1038/s41588-019-0374-2

^9^

Cussins, J., & Lowthorp, L. (2018). Germline modification and policymaking: The relationship between mitochondrial replacement and gene editing. *New Bioethics, 24*(1), 74–94, p. 83.10.1080/20502877.2018.144340929529982

^10^

Savulescu, J. (2018). The fundamental ethical flaw in Jiankui He’s alleged gene editing experiment. *Ethics in the News* (blog). Published November 28. Retrieved from http://blog.practicalethics.ox.ac.uk/2018/11/the‐fundamental‐ethical‐flaw‐in‐jiankui‐hes‐alleged‐gene‐editing‐experiment/

^11^

Parfit, D. (1984). *Reasons and persons*. Oxford, U.K.: Clarendon Press; Heyd, D. (2014). Parfit on the non‐identity problem, again. *Law Ethics Human Rights, 8* (1), 1–20.

^12^

Boonin, D. (2014). *The non‐identity problem and the ethics of future people*. Oxford Scholarship Online: November 2014. 10.1093/acprof:oso/9780199682935.001.0001, p. 2. We will restrict ourselves to what Boonin calls the direct version of the non‐identity problems. The indirect version, in which our actions create different persons in the far distant future, pose different problems that we will not tackle in this paper.

^13^

Roberts, M. A., & Wasserman, D. T. (2009). Harming future persons: Introduction. In M. A. Roberts & D. T. Wasserman (Eds.), *Harming future persons: Ethics, genetics and the nonidentity* *problem*. Dordrecht, Germany: Springer, pp. xix‐xx.

^14^

Boonin, op. cit. note 12, p. 14.

^15^

Savulescu, J. (2002). Deaf lesbians, ‘designer disability’, and the future of medicine. *British Medical Journal, 325*(7367), 771–773.10.1136/bmj.325.7367.771PMC112427912364309

^16^

Savulescu, J., & Kahane, G. (2017). Understanding procreative beneficence In L. Francis (Ed.), *The Oxford handbook of reproductive ethics*, Chapter 26 (pp. 592–622). Oxford, U.K.: Oxford University Press.

^17^

Savulescu, J., & Singer, P. (2019). An ethical pathway for gene editing. *Bioethics 33*(2) 221–222.10.1111/bioe.1257030695116

^18^

Raposo V. L. (2019). The first Chinese edited babies: A leap of faith in science. *JBRA Assisted Reproduction, 23*(3), 197–199.10.5935/1518-0557.20190042PMC672438831436399

^19^

Glass, W. G, McDermott, D. H., Lim, J. K., Lekhong, S., Yu, S. F., Frank, W. A. … Murphy, P. M. (2006). CCR5 deficiency increases risk of symptomatic West Nile virus infection. *Journal of Experimental Medicine, 203*, 35–40.10.1084/jem.20051970PMC211808616418398

^20^

Qiu, R., Lei, R., & Zhai, X. (2019) Lessons from the He Jiankui incident. *Issues in Science and Technology, 35*(4), 20–22.

^21^

DeGrazia, D. (2005). *Human identity and bioethics*. Cambridge: Cambridge University Press; Parfit, op. cit. note 11.

^22^

Persson, on the other hand, has defended that gene therapy interventions are, in principle, identity‐preserving (Persson, I. (1995). Genetic therapy, identity and the person‐regarding reasons. *Bioethics, 9*(1), 16–31).10.1111/j.1467-8519.1995.tb00298.x11653030

^23^

McMahan, J. (1998). Wrongful life: Paradoxes in the morality of causing people to exist. In J. L. Coleman & C. W. Morris (Eds.). *Rational commitment and social justice: Essays for Gregory Kavka* (pp. 208–249). Cambridge, U.K.: Cambridge University Press.

^24^

Savulescu, J. (2011). Genetically modified animals. In T. Beauchamp & R. Frey (Eds.), *The Oxford handbook of animal ethics*, Chapter 24 (pp. 641–670). Oxford, U.K.: Oxford University Press.

^25^

‘Without antiretroviral intervention, the reported rates of HIV vertical transmission are 15‐25% in developed countries, and as high as 25‐40% according to studies carried out in African or Asian countries’: Barreiro, P., Duerr, A., Beckerman, K., & Soriano, V. (2006). Reproductive options for HIV‐serodiscordant couples. *AIDS Reviews, 8*, 158‐170, p. 164.17078486

^26^

Brock, D. W. (1995). Genetic harms and the non‐identity problem. *Bioethics 9* (3/4), 269–275; Feinberg, J. (1992). Wrongful life and the counterfactual element in harming. In *Freedom and* *fulfillment: Philosophical essays* (pp. 3–36). Princeton, NJ: Princeton University Press; Parfit, op. cit. note 11.

^27^

Roberts & Wasserman, op. cit. note 13, p. xxi. A related solution to the non‐identity problem, sometimes considered complementary to impersonal views, is the so‐called ‘impartiality principle’. This principle, defended by, among others, Lillehammer (Lillehammer, H. (2009). Reproduction, partiality and the non‐identity problem. In M. A. Roberts & D. T. Wasserman (Eds.), *Harming future persons: Ethics, genetics and the nonidentity* *problem* (pp. 231–247). Dordrecht, Germany: Springer) requires me to put as much weight on the interests of everyone else as I put on my own interests—everyone being also possible people. This reference to possible people carries many theoretical and practical problems (see Boonin, op. cit. note 12, pp. 158–160).

^28^

Savulescu & Singer, op. cit. note 17.

^29^

Roberts & Wasserman, op. cit. note 13, p. xxi.

^30^

Parfit, op. cit. note 11, p. 360.

^31^

Savulescu & Kahane, op. cit. note 16.

^32^

Magnusson, E. (2019). Children’s rights and the non‐identity problem. *Canadian Journal of Philosophy,* *49*(5), 580–605, p. 590.

^33^

Boonin, op. cit. note 12, p. 116.

^34^

Ibid.

^35^

Ibid: 141.

^36^

Wasserman, D. (2005). The non‐identity problem, disability, and the role morality of prospective parents. *Ethics, 116*(1), 132–152.10.1086/45436916578954

^37^

Harman, E. (2004). Can we harm and benefit in creating? *Philosophical Perspectives, 18*, 89–113; Shiffrin, S. V. (1999). Wrongful life, procreative responsibility, and the significance of harm. *Legal Theory, 5*, 117–148.

^38^

Harman, op. cit. note 33, p. 93.

^39^

Ibid: 98.

^40^

This argument seems to resemble the one made by Harman about a Nazi prisoner whose experience in a concentration camp benefited him. Harman states that, even if the Nazis had a ‘rare insight into the causal consequences that would occur (…) their actions would still have been impermissible’ (Harman, op. cit., p. 101). However, we believe that in this type of discussion concrete cases matter, and that the Bezos case might be importantly different from the Nazi prisoner’s case. We believe that in the Nazi prisoner example it is simply impossible to imagine and believe that the Nazis had the intention of benefiting. Nazism was a systemic structure to oppress and harm people. In the Bezos case, however, one can perfectly imagine him going crazy; and the 10 million compensation is a very concrete beneficial consequence which might make the act justifiable [Savulescu, J. (2001) Taking the plunge. *New Scientist, 2280*, 50; Anomaly, J., & Savulescu, J. (2019). Compensation for cures: Why we should pay a premium for participation in ‘challenge studies’. *Bioethics, 33*(7), 792–797].10.1111/bioe.12596PMC677322131135070

^41^

Boonin, op. cit. note 12, p. 181.

^42^

Narveson, J. (1973). Moral problems of population. *Monist, 57*(1), 62–86, p. 80.10.5840/monist19735713411661014

^43^

Roberts, M. (1998). *Child versus* *childmaker: Future persons and present duties in ethics and the law*. Oxford, U.K.: Rowman and Littlefield; Hull, R. J. (2006). Cheap listening? Reflections on the concept of wrongful disability. *Bioethics, 20*(2), 55–63; Boonin, op. cit. note 12.10.1111/j.1467-8519.2006.00477.x16770864

^44^

Boonin, op. cit. note 12, p. 191.

^45^

Ibid: 192.

^46^

Ibid.

^47^

Savulescu, J. (1998). The present‐aim theory: A submaximizing theory of reasons? *Australasian Journal of Philosophy, 76*(2), 229–243.

^48^

This conclusion is something Boonin is fully aware of: ‘While every other solution to the nonidentity problem therefore tends in the direction of less reproductive moral freedom, my solution tends in the direction of more.’ Boonin, op. cit. note 12, pp. 215–216.

^49^

We thank one anonymous reviewer for this example.

^50^

Cohen, J. (2019, August 1). Did CRISPR help—or harm—the first‐ever gene‐edited babies? *Science*. doi: 10.1126/science.aay9569

^51^

Cohen, J. (2018). ‘I feel an obligation to be balanced’. Noted biologist comes to defense of gene editing babies, *Science*. https://www.sciencemag.org/news/2018/11/i‐feel‐obligation‐be‐balancednoted‐biologist‐comes‐defense‐gene‐editing‐babies [accessed Feb 28, 2019].

^52^

Wrigley, A., Wilkinson, S., & Appleby, J. B. (2015) Mitochondrial replacement: Ethics and identity. *Bioethics, 29*, 9631‐9638.10.1111/bioe.12187PMC462070326481204

^53^

Ibid: 635.

^54^

Rulli, T. (2017). The mitochondrial replacement ‘therapy’ myth. *Bioethics, 31*(5), 368–374, p. 372.10.1111/bioe.1233228039887

^55^

Ibid.

^56^

Ibid: 373.

^57^

We thank an anonymous reviewer for these examples.

^58^

Rulli, op. cit. note 48, p. 369.

^59^

Woollard, F. (2012). Have we solved the non‐identity problem? *Ethical Theory and Moral Practice, 15*(5), 677–690, p. 688.

^60^

McMahan, J. (2013). Causing people to exist and saving people’s lives. *Journal of Ethics, 17*(1), 5–35, p. 34.

^61^

Rulli, op. cit. note 48, p. 374.

